# Real-World Experience With a Novel Intravascular Hertz Contact Lithotripsy Balloon in Severely Calcified Coronary Lesions

**DOI:** 10.1016/j.jacadv.2026.102958

**Published:** 2026-07-03

**Authors:** Rodolfo Caminiti, Alfonso Ielasi, Giampiero Vizzari, Andrea Marrone, Roberto Licordari, Gabriele Monciino, Gabriele Carciotto, Dario Pellegrini, Mariano Pellicano, Antonio Micari

**Affiliations:** a U.O. Cardiologia Ospedaliera, IRCCS Ospedale Galeazzi Sant'Ambrogio, Milan, Italy; b Cardiology Unit, Department of Clinical and Experimental Medicine, University of Messina, Messina, Italy

**Keywords:** coronary calcification, Hertz contact lithotripsy, intravascular lithotripsy, optical coherence tomography, percutaneous coronary intervention

Severe coronary calcification remains a major determinant of procedural complexity and adverse outcomes in percutaneous coronary intervention (PCI), particularly in older adults with multiple comorbidities.^1^ Inadequate lesion preparation in this setting is strongly associated with suboptimal stent expansion, malapposition, and an increased risk of restenosis and stent thrombosis.^2^ Although multiple plaque-modifying technologies are currently available, including atherectomy-based approaches (rotational and orbital ablation, laser atherectomy) and intravascular lithotripsy (IVL), each modality has intrinsic limitations related to deliverability, lesion morphology, and procedural risk.^3^ The LithiX balloon (Elixir Medical Corporation) is a novel lesion preparation device designed to enhance coronary calcium modification while preserving a standard balloon-based workflow.^4^ The technology is based on the Hertz physical phenomenon, whereby mechanical energy is generated and transmitted through brief, localized pressure waves during balloon inflation.^5^ Specifically, multiple metallic hemispheres distributed over the surface of the balloon act as energy emitters; when the balloon is inflated, these elements generate focal mechanical impulses that are directly transmitted to the vessel wall. The delivered mechanical energy propagates through the calcified plaque, inducing microfractures and fragmentation of both superficial and deep calcium, thereby increasing vessel compliance. Unlike conventional energy-based intravascular balloon systems, the LithiX balloon does not require an external generator or dedicated console, since mechanical energy generation through Hertzian contact stress is intrinsically linked to balloon inflation. The device is also available in small balloon profiles and incorporates a folding design that may enhance trackability and facilitate lesion crossing, preserving a workflow comparable to standard balloon-based lesion preparation. In this context, LithiX may represent a practical alternative within the contemporary calcium-modification armamentarium, particularly for selected long or mid-distal calcified lesions in which deliverability and procedural simplicity are key factors.

However, real-world data on its performance and mechanistic effects assessed by intracoronary imaging remain limited. We report an initial clinical experience focusing on feasibility, safety, and optical coherence tomography (OCT)-defined calcium modification following LithiX balloon use in patients with severely calcified coronary lesions.

In this early real-world experience conducted at 2 centers, we retrospectively evaluated 16 consecutive older adults (mean age 76 ± 7 years) with chronic coronary syndrome undergoing PCI for de novo heavily calcified coronary lesions over a 2-month period. Enrolled patients had a high prevalence of hypertension and dyslipidemia, diabetes mellitus in 5/16 patients (31.3%), and a history of prior PCI in 5/16 patients (31.3%). All procedures were performed under OCT guidance before and after lesion preparation to characterize calcium morphology and assess the acute mechanical effects of the LithiX balloon. Target lesions were selected for LithiX balloon treatment at the operator’s discretion after baseline OCT assessment, mainly in the presence of severe superficial or deep calcification, long calcified segments, circumferential calcium, and/or calcium nodules. Overall, lesions were predominantly located in the mid segments of major epicardial vessels. Preintervention OCT demonstrated extensive calcific burden in all lesions, with predominantly circumferential calcification in with predominantly circumferential calcification, defined as calcium arc >180°, observed in 10/16 lesions (62.5%), whereas calcium nodules were identified in 5/16 lesions (31.3%). The mean calcium length was 25.7 ± 5.7 mm, and the minimum lumen area at baseline was 2.5 ± 0.9 mm^2^. The LithiX balloon was selected as the primary calcium modification strategy in all cases and was successfully delivered and expanded in 15 of 16 patients with a technical success rate of 93.8% (95% CI: 69.8%-99.8%). Overall procedural success, defined as successful lesion preparation and drug-eluting stent (DES) implantation with an excellent final angiographic result and no major angiographic complications, was achieved in 16/16 patients (95% CI: 79.4% to 100%). In 1 patient, persistent inadequate lesion modification after LithiX treatment in the setting of extreme calcific burden prompted adjunctive use of a Shockwave IVL balloon, with the aim of providing additional circumferential acoustic-pulse calcium modification before DES implantation. Among successfully treated lesions, post-LithiX OCT demonstrated effective calcium modification with visible fractures and fragmentation. (Figure 1) Following lesion preparation, all successfully treated patients underwent DES) implantation with excellent acute angiographic results and no complications (eg coronary perforation, abrupt vessel closure, slow-flow, or no-reflow phenomena). Final OCT confirmed optimal stent deployment across all calcium morphologies, showing a final DES cross-sectional area of 7.0 ± 1.6 mm^2^, with a net lumen gain of 4.0 ± 1.7 mm^2^ in eccentric and 5.1 ± 1.8 mm^2^ in concentric calcified lesions.

**Figure 1:**
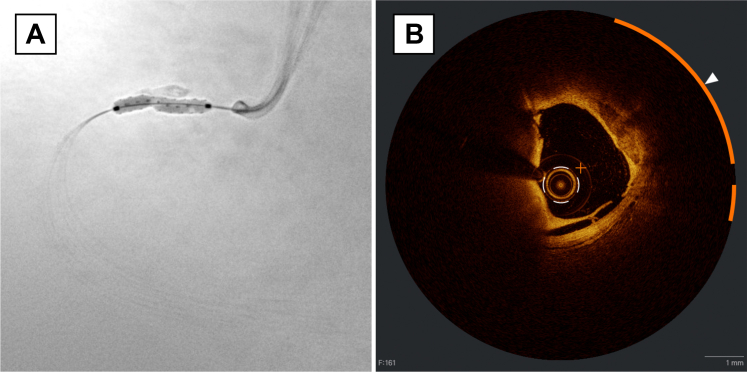
Angiographic and Optical Coherence Tomography Assessment of LithiX Balloon–Mediated Calcium Modification (A) Angiographic view showing the LithiX balloon positioned across a severely calcified coronary lesion during initial inflation; (B) Post–LithiX balloon OCT image demonstrating multiple calcium fractures and plaque fragmentation.

In this initial two-center experience, the LithiX IVL balloon demonstrated a favorable safety profile and effective acute calcium modification, translating into satisfactory lesion preparation and optimal stent expansion in patients with severely calcified coronary lesions. Although limited by the small sample size and short-term follow-up, these findings suggest that LithiX may represent a practical and potentially cost-effective addition to the contemporary armamentarium for plaque modification, complementing existing technologies in appropriately selected lesions (eg, long calcified lesions located in mid-distal segments). Larger prospective studies are warranted to further define long-term outcomes.

## Funding support and author disclosures

The authors have reported that they have no relationships relevant to the contents of this paper to disclose.
